# Albumin infusion may decrease the mortality of hypoalbuminemia patients with severe acute pancreatitis: a retrospective cohort study

**DOI:** 10.1186/s12876-023-02801-8

**Published:** 2023-06-05

**Authors:** Huiting Xu, Jianhua Wan, Wenhua He, Yong Zhu, Hao Zeng, Pi Liu, Jing Liu, Liang Xia, Fen Liu, Yin Zhu, Youxiang Chen, Nonghua Lu

**Affiliations:** 1 Department of Gastroenterology, The First Affiliated Hospital of Nanchang University, 17 Yongwaizheng Street, Nanchang, 330006 Jiangxi PR China; 2 Department of Pharmacy, The First Affiliated Hospital of Nanchang University, Nanchang, PR China; 3 Department of Intensive Care Unit, The First Affiliated Hospital of Nanchang University, Nanchang, PR China

**Keywords:** Severe acute pancreatitis, Albumin infusion, Hypoalbuminemia, Mortality, Prognosis

## Abstract

**Background:**

At present, the relationship between severe acute pancreatitis (SAP) and albumin infusion is not clear. We aimed to identify the impact of serum albumin on the prognosis of SAP and the association between albumin infusions and mortality for hypoalbuminemia patients.

**Methods:**

This was a retrospective cohort study that analyzed 1000 patients with SAP who were admitted to the First Affiliated Hospital of Nanchang University between January 2010 and December 2021 using data from a prospectively maintained database. Multivariate logistic regression analysis was conducted to reveal the relationship between serum albumin within 1 week after admission and poor prognosis of SAP. Propensity score matching (PSM) analysis was adopted to evaluate the effect of albumin infusion for hypoalbuminemia patients with SAP.

**Results:**

The prevalence of hypoalbuminemia (≤ 30 g/L) was 56.9% within 1 week after admission. Multivariate logistic regression identified that age (OR: 1.02; 95% CI: 1.00-1.04; P = 0.012), serum urea (OR: 1.08; 95% CI: 1.04–1.12; P < 0.001), serum calcium (OR: 0.27; 95% CI: 0.14–0.50; P < 0.001), lowest albumin level within 1 week after admission (OR: 0.93; 95% CI: 0.89–0.97; P = 0.002), and APACHE II score ≥ 15 (OR: 1.73; 95% CI: 1.19–2.51; P = 0.004) were independently associated with mortality. The PSM analysis demonstrated that mortality (OR: 0.52, 95% CI: 0.29–0.92, P = 0.023) was less common in albumin-infused than non-albumin-infused hypoalbuminemia patients. In subgroup analyses, doses > 100 g within 1 week after admission for hypoalbuminemia patients with albumin infusions was associated with lower mortality than doses ≤ 100 g (OR: 0.51, 95% CI: 0.28–0.90, P = 0.020).

**Conclusions:**

Hypoalbuminemia in early-stage SAP is significantly related to poor prognosis. However, albumin infusions could significantly decrease mortality in hypoalbuminemia patients with SAP. Additionally, infusing sufficient albumin within a week after admission may decrease mortality in hypoalbuminemia patients.

**Supplementary Information:**

The online version contains supplementary material available at 10.1186/s12876-023-02801-8.

## Introduction

Acute pancreatitis (AP) is a major gastrointestinal disease that has an increasing incidence and generally requires acute hospitalization [1]. Approximately 10–20% of patients develop severe acute pancreatitis (SAP) with persistent organ failure, which is associated with a significant mortality rate of 20–40% [2–5]. Therefore, it is critical to identify high-risk patients and provide effective treatments, which could significantly decrease the mortality of SAP patients. Existing scoring systems and laboratory parameters, such as the Bedside Index of Severity in Acute Pancreatitis (BISAP) [6], the Acute Physiology and Chronic Health Examination (APACHE) II [7], blood urea nitrogen [8], hematocrit [9], and serum calcium [10], are used to diagnose and evaluate the severity of AP.

Albumin is the most abundant plasma protein; it is produced by the liver and is the primary factor in the maintenance of colloidal osmotic pressure [11]. Hypoalbuminemia is commonly noticed in patients with sepsis, kidney failure, nephrotic syndrome, cancer, decompensated liver cirrhosis and surgical operation or elderly hospitalized patients [12–17]. Similarly, this phenomenon can occur in acute pancreatitis, especially SAP. Some studies have demonstrated that serum albumin as a useful biomarker might be a significant tool in predicting adverse outcomes of AP, particularly in predicting persistent organ failure and death [18–20]. However, few studies have predicted hypoalbuminemia and outcomes of SAP.

Human serum albumin treatments are mainly used for hypoalbuminemia correction and fluid resuscitation in critically ill patients, such as those with sepsis and liver cirrhosis patients [21–24]. However, it is still controversial whether the use of albumin can improve the clinical outcomes. It is unclear whether albumin infusion could improve the clinical prognosis of SAP with hypoalbuminemia, although SAP has many similarities to sepsis syndrome and septic shock [25].

Therefore, we aimed to further verify the impact of hypoalbuminemia on the prognosis of SAP patients and the association between albumin infusions and mortality for hypoalbuminemia patients in this retrospective study.

## Method

### Study design and participants

This was a retrospective cohort study that analyzed first-episode SAP patients hospitalized in the Department of Gastroenterology of the First Affiliated Hospital of Nanchang University between January 2010 and December 2021. The ethics committee of The First Affiliated Hospital of Nanchang University reviewed and approved this study (No. 2,011,001).

**Fig. 1:**
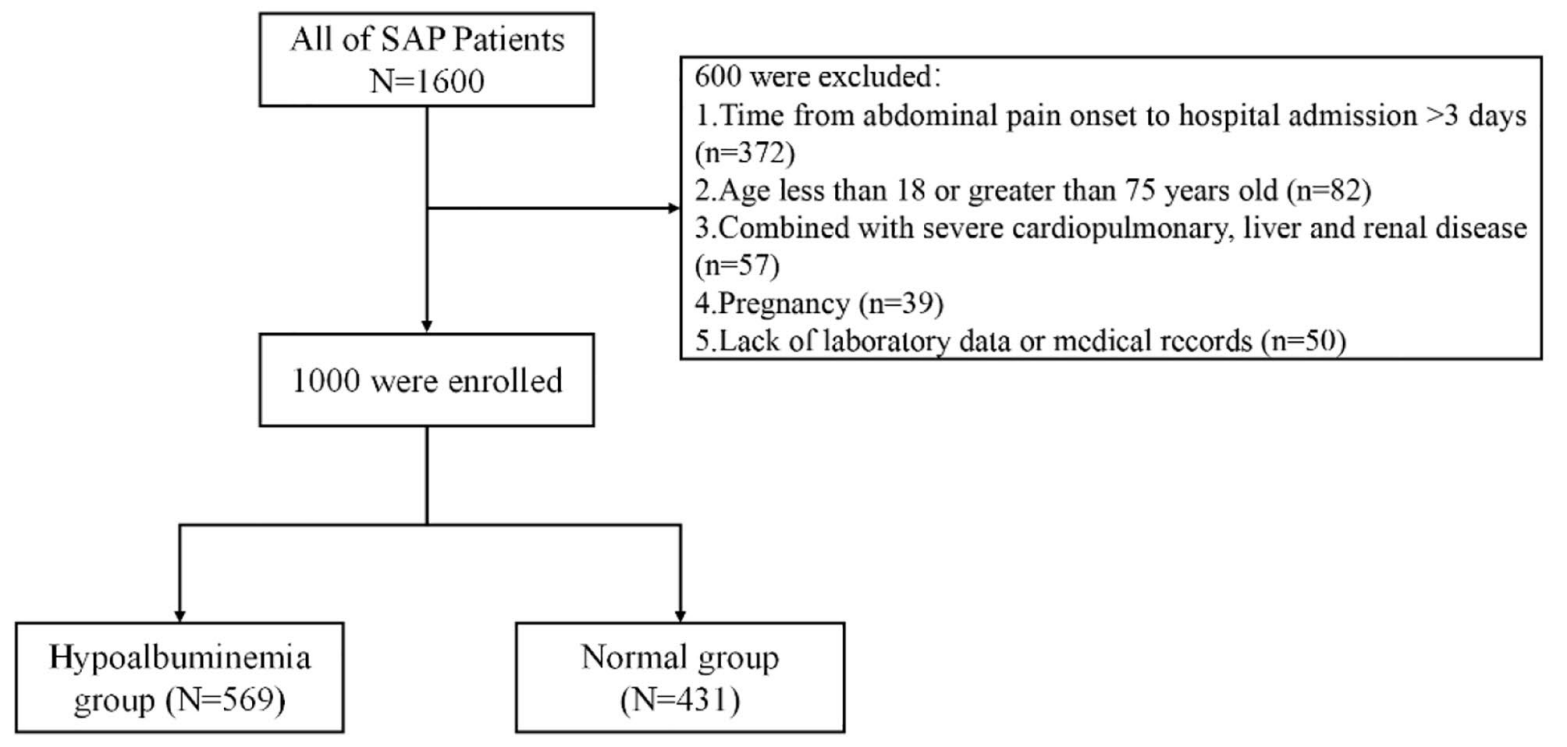
Flow diagram of patient inclusion and exclusion

The diagnosis and classification of acute pancreatitis are in accordance with the 2012 revision of the Atlanta classification, which is well recognized [5]. The exclusion criteria were as follows: (1) time from abdominal pain onset to hospital admission > 3 days; (2) age less than 18 or greater than 75 years old; (3) severe cardiopulmonary, liver and renal disease; (4) SAP during pregnancy and (5) lack of laboratory data or medical records. All data were collected from a prospectively maintained database of 8012 AP patients where we extracted 1600 SAP patients. Age, sex, BMI, some biochemical variables and severity scores were correctly recorded within 24 h of admission. In addition, the serum albumin levels were measured 3–5 times within 1 week after hospitalization according to the patient’s condition, and the lowest albumin level was included. Then, an albumin level of 30 g/L was used as the cutoff value for the grouping, and this cutoff value is similar to previous studies [26, 27]. Hypoalbuminemia was defined as a serum albumin level ≤ 30 g/L. Based on the lowest albumin level within 1 week after admission, the patients were divided into hypoalbuminemia and normal groups. The process of selection was shown in Fig. 1. The primary treatment methods for AP patients include early fluid resuscitation, nutrition support and analgesia, as well as treatment for etiology and complications [4, 28–30].

### Definitions

Sepsis is defined as systemic inflammatory response syndrome caused by definite or suspected infection [31]. Infected pancreatic necrosis (IPN) was defined by at least one of the following findings: presence of gas bubbles in the pancreas and/or in the peripancreatic tissue on a CT examination, a positive smear staining or culture result of samples obtained by imaging-guided fine needle aspiration, and/or during the first intervention (either by drainage or by surgery) [5, 32]. Shock was defined as a systolic blood pressure < 90mmHg [28]. Abdominal compartment syndrome (ACS) was defined as a sustained intra-abdominal pressure (IAP) > 20 mmHg with evidence of organ dysfunction [33]. The outcomes also included the length of intensive care unit (ICU) stay, length of hospital stay, and total hospital costs, which were all defined during the whole hospitalization.

### Outcomes

In this study, the primary outcome was in-hospital mortality of SAP. The secondary outcomes included sepsis, IPN, shock, ACS, persistent organ failure, the rate of mechanical ventilation and hemofiltration, length of ICU stay, length of hospital stay and total hospital costs.

### Statistical analysis

Continuous data are expressed as means ± standard deviations or medians (interquartile ranges) and were analyzed using the student’s t test or Mann–Whitney U test, as appropriate. Categorical data are presented as absolute numbers and percentages and were analyzed using the chi-square test. The factors related to mortality in the univariable models (P < 0.05) were brought into the multivariable models to further identify the independent risk factors related to mortality.

A caliper value of 0.02 was used for 1:1 nearest neighbor matching by propensity scores without replacement to minimize the confounding effects. Variables (age, sex, BMI, etiology, initial value of blood albumin, initial value of blood urea, initial value of blood creatinine, initial value of blood calcium, lowest albumin level within 1 week after admission, SIRS score, and APACHE II score, mechanical ventilation, hemofiltration, persistent multiple organ failure, persistent single organ failure and IPN) were selected for propensity score matching (PSM). Mann–Whitney U test and χ^2^ test or Fisher’s exact test were used to assess the comparability of baseline characteristics in the matched groups. Similarly, comparison of the mortality rate during hospitalization was analyzed using the χ^2^ test or Fisher’s exact test in the matched cohorts.

The odds ratio (OR) and 95% confidence intervals (CI) were measured. A two-tailed P value < 0.05 was considered statistically significant. Statistical analyses were performed using SPSS software (v25.0; SPSS Inc., Chicago, IL, USA).

## Results

### Baseline characteristics of the patients in the hypoalbuminemia group and the normal group

There were 1600 patients with SAP in total, but 1000 patients met the inclusion and exclusion criteria in this study (Fig. 1). A total of 633 (63.3%) were male, and they had a median age of 51.0 (IQR: 21.0) years. The median value of serum albumin within 24 h of admission was 32.9 g/L (IQR: 7.1). However, the median value of the lowest albumin level within 1 week after admission was 30.0 g/L (IQR: 5.3). And the median time from admission to sample the lowest albumin level during the first week was 3 days (IQR:3.0). The overall prevalence of hypoalbuminemia (≤ 30 g/L) was 56.9% (569/1000). Therefore, there were 431 SAP patients in the normal group and 569 SAP patients in the hypoalbuminemia group. The demographic, past history, laboratory findings and clinical scoring systems at hospital admission of the patients in the different groups are shown in Table 1. Older age (P < 0.001) and decreased BMI (P < 0.001) were associated with hypoalbuminemia. The patients with normal albumin levels were more likely to be male than those with hypoalbuminemia (70.5% vs. 57.8%; P < 0.001). The etiological distribution of SAP was significantly different between groups, with hyperlipidemic pancreatitis being less common in the hypoalbuminemia group (35.3% vs. 47.6%; P < 0.001). No significant difference was observed among the patients in the different groups with respect to hypertension, diabetes mellitus, white blood count and SIRS score ≥ 2. Compared with the normal group (N = 431), the hypoalbuminemia group (N = 569) had significant differences in hematocrit (44.4 (10.2) vs. 43.2 (11.2), P = 0.023), serum albumin (35.6 (5.9) vs. 30.2 (6.2), P < 0.001), serum urea (7.2 (4.9) vs. 9.2 (7.7), P < 0.001), serum creatinine (77.0 (57.2) vs. 95.0 (130.0), P < 0.001), serum calcium (2.0 (0.4) vs. 1.8 (0.4), P < 0.001) and APACHE II score ≥ 15 (76 (17.6%) vs. 133 (23.4%), P < 0.001).

**Table 1:** Comparison of baseline characteristics in the 1000 subjects by the lowest albumin level within 1 week after admission

Variables	**Hypoalbuminemia group**	Normal group	P
	**(N = 569)**	**(N = 431)**	
Age, years, IQR	53.0(21.0)	47.0(22.0)	< 0.001
Sex (male), n(%)	329(57.8)	304(70.5)	< 0.001
BMI, kg/m^2^, IQR	24.1(3.9)	25.1(4.5)	< 0.001
Etiology, n(%)			< 0.001
Biliary	294(51.7)	169(39.2)	
Hyperlipidemia	201(35.3)	205(47.6)	
Alcoholic	32(5.6)	26(6.0)	
Other	42(7.4)	31(7.2)	
Hypertension, n(%)	151(26.5)	99(23.0)	0.197
Diabetes mellitus, n(%)	76(13.4)	71(16.5)	0.168
Laboratory data at admission, IQR			
White blood count, × 10^9^/L	14.0(7.8)	14.0(7.1)	0.212
Hematocrit, %	43.2(11.2)	44.4(10.2)	0.023
Serum albumin, g/L	30.2(6.2)	35.6(5.9)	< 0.001
Serum urea, mmol/L	9.2(7.7)	7.2(4.9)	< 0.001
Serum creatinine, µmol/L	95.0(130.0)	77.0(57.2)	< 0.001
Serum calcium, mmol/L	1.8(0.4)	2.0(0.4)	< 0.001
Severity scores at admission, n(%)			
SIRS score ≥ 2	466(81.9)	332(77.0)	0.058
APACHE≥score ≥ 15	133(23.4)	76(17.6)	0.027

IQR, Inter Quartile Range; BMI, Body Mass Index; SIRS, Systemic Inflammatory Response Syndrome; APACHE, Acute Physiology and Chronic Health Evaluation

### Comparison of the primary and secondary outcomes in the different groups

Low serum albumin levels were associated with an increased risk of adverse clinical outcomes. For the primary outcome, there was a significant difference in death (18.1% vs. 31.6%, P < 0.001). For some of the secondary outcomes, significant differences were observed among the patients in the two groups with respect to sepsis (7.2% vs. 15.1%, P < 0.001), IPN (11.6% vs. 17.4%, P = 0.011), ACS (6.3% vs. 11.1%, P < 0.001), persistent multiple organ failure (19.3% vs. 32.2%, P < 0.001), persistent renal failure (16.0% vs. 28.1%, P < 0.001), persistent cardiovascular failure (13.2% vs. 18.3%, P = 0.031), need for mechanical ventilation (24.6% vs. 46.4%, P < 0.001) and hemofiltration (9.5% vs. 15.3%, P = 0.007). Additionally, a prolonged hospital stay (13.0 (11.0) vs. 18.0 (19.0); P < 0.001) and ICU stay (4.0 (10.0) vs. 9.0 (15.0); P < 0.001) and increased hospitalization costs (4.3 (6.3) vs. 7.6 (11.5); P < 0.001) were noticed in the hypoalbuminemia group compared with the normal group. However, for the other secondary outcomes, such as shock and single respiratory failure, there was no significant difference (Table 2).

**Table 2:** Primary and secondary outcomes according to the different groups by the lowest albumin level within 1 week after admission

Variables	Hypoalbuminemia group	Normal group	*P*
	(N = 569)	(N = 431)	
Primary outcome			
Death, n(%)	180(31.6)	78(18.1)	< 0.001
Secondary outcomes			
Sepsis, n(%)	86(15.1)	31(7.2)	< 0.001
IPN, n(%)	99(17.4)	50(11.6)	0.011
Shock, n(%)	95(16.7)	58(13.5)	0.159
ACS, n(%)	63(11.1)	27(6.3)	< 0.001
Multiple persistent organ failure, n(%)	183(32.2)	83(19.3)	< 0.001
Single persistent organ failure, n(%)			
Respiratory	515(90.5)	402(93.3)	0.117
Renal	160(28.1)	69(16.0)	< 0.001
Cardiovascular	104(18.3)	57(13.2)	0.031
Mechanical ventilation, n(%)	264(46.4)	106(24.6)	< 0.001
Hemofiltration, n(%)	87(15.3)	41(9.5)	0.007
Hospital stay, days, IQR	18.0(19.0)	13.0(11.0)	< 0.001
Hospital stay in ICU, days, IQR	9.0(15.0)	4.0(10.0)	< 0.001
Hospital total costs, million yuan, IQR	7.6(11.5)	4.3(6.3)	< 0.001

IQR, Inter Quartile Range; IPN, Infected Pancreatic Necrosis; ACS, Abdominal Compartment Syndrome

### The lowest albumin level within 1 week after admission as an independent prognostic factor for mortality

To further investigate the association between the lowest albumin level and the mortality rate in SAP patients, we first conducted a univariate logistic regression analysis model, which consisted of age, sex, BMI, etiology, hypertension, diabetes mellitus, white blood count, hematocrit, initial value of blood albumin, initial value of blood urea, initial value of blood creatinine, initial value of blood calcium, lowest albumin level within 1 week after admission, SIRS score ≥ 2 and APACHE II score ≥ 15. Next, the factors associated with mortality in the unadjusted models (P < 0.05) were included in the multivariate logistic regression analysis model. These results are detailed in Table 3. Multivariate logistic regression identified that age (OR: 1.02; 95% CI: 1.00-1.04; P = 0.012), serum urea (OR: 1.08; 95% CI: 1.04–1.12; P < 0.001), serum calcium (OR: 0.27; 95% CI: 0.14–0.50; P < 0.001), the lowest albumin level within 1 week after admission (OR: 0.93; 95% CI: 0.89–0.97; P = 0.002), and APACHE II score ≥ 15 (OR: 1.73; 95% CI: 1.19–2.51; P = 0.004) were independently associated with mortality.

**Table 3:** Univariate and multivariate logistic regression analyses of risk factors for mortality

Variables	Univariate analysis Odds ratio (95%CI)	P	Multivariate analysis Odds ratio (95%CI)	P
Age, years, IQR	1.03(1.02–1.04)	< 0.001	1.02(1.00-1.04)	0.012
Sex (male), n(%)	0.78(0.58–1.04)	0.090		
BMI, kg/m^2^, IQR	0.56(0.94–1.02)	0.348		
Etiology, n(%)				
Biliary	Ref		Ref	
Hyperlipidemic	0.66(0.48–0.90)	0.008	1.0(0.52–1.81)	0.919
Alcoholic	1.03(0.57–1.88)	0.926	0.70(0.36–1.35)	0.284
Other	1.14(0.67–1.95)	0.627	1.0(0.42–2.34)	0.991
Hypertension, n(%)	1.33(0.97–1.82)	0.080		
Diabetes mellitus, n(%)	1.09(0.73–1.62)	0.672		
Laboratory data at admission, IQR				
White blood count, × 10^9^/L	1.02(0.99–1.05)	0.090		
Hematocrit, %	0.99(0.97-1.00)	0.116		
Serum albumin, g/L	0.92(0.89–0.95)	< 0.001	1.03(0.99–1.08)	0.165
Serum urea, mmol/L	1.13(1.11–1.17)	< 0.001	1.08(1.04–1.12)	< 0.001
Serum creatinine, µmol/L	1.01(1.00-1.01)	< 0.001	1.00(0.99-1.00)	0.331
Serum calcium, mmol/L	0.19(0.12–0.31)	< 0.001	0.27(0.14–0.50)	< 0.001
Serum albumin _min_, g/L	0.90(0.87–0.93)	< 0.001	0.93(0.89–0.97)	0.002
SIRS score ≥ 2, n(%)	1.47(1.01–2.14)	0.045	1.16(0.75–1.80)	0.506
APACHE≥score ≥ 15, n(%)	3.12(2.26–4.3)	< 0.001	1.73(1.19–2.51)	0.004

IQR, Inter Quartile Range; BMI, Body Mass Index; Serum albumin _min_, the lowest albumin level within 1 week after admission; SIRS, Systemic Inflammatory Response Syndrome; APACHE, Acute Physiology and Chronic Health Evaluation

### Propensity score matching (PSM) analysis for hypoalbuminemia patients

For SAP patients with hypoalbuminemia, a PSM analysis was further performed to explore the relevant effect of human serum albumin infusion on patient outcomes. There were 569 patients in total divided into two groups (with albumin infusions group and without albumin infusions group) according to whether albumin was infused during hospitalization. Ultimately, 260 patients remained for further analysis (130 in each cohort) in totally 569 hypoalbuminemia patients. The baseline characteristics were balanced in the matched groups, which was shown in Table 4. Before PSM, there was no difference in mortality between the two groups (OR:0.89, 95%CI:0.60–1.33, P = 0.562). Nevertheless, after PSM, the hypoalbuminemia patients with albumin infusions had significant differences in mortality compared to those without albumin infusions (OR:0.52, 95%CI:0.29–0.92, P = 0.023). However, in the albumin normal group, there was no difference in whether albumin was infused or not when the same matching principle was adopted (OR:0.58, 95%CI:0.29–1.16, P = 0.121) (Table 5).

**Table 4:** Baseline situation of the two groups with and without albumin infusions before and after PSM analysis in hypoalbuminemia patients

Variables	Before Matching			After Matching		
	With albumin infusions	Without albumin infusions	*P*	With albumin infusions	Without albumin infusions	*P*
	(N = 423)	(N = 146)		(N = 130)	(N = 130)	
Age, years, IQR	52.0(21.0)	55.0(21.0)	0.171	53.0(19.0)	55.0(21.0)	0.634
Sex (male), n(%)	249(58.9)	80(54.8)	0.390	73(56.2)	69(53.1)	0.618
BMI, kg/m^2^, IQR	24.1(3.6)	23.9(4.3)	0.399	24.1(3.5)	23.9(4.5)	0.741
Etiology, n(%)			0.348			0.479
Biliary	210(49.6)	84(57.5)		68(52.3)	79(60.8)	
Hyperlipidemia	157(37.1)	44(30.1)		45(34.6)	36(27.7)	
Alcoholic	23(5.4)	9(6.2)		8(6.2)	9(6.9)	
Other	33(7.8)	9(6.2)		9(6.9)	6(4.6)	
Hypertension, n(%)	116(27.4)	35(24.0)	0.416	32(24.6)	28(21.5)	0.556
Diabetes mellitus, n(%)	59(13.9)	17(11.6)	0.480	14(10.8)	13(10.0)	0.839
Laboratory data at admission, IQR						
White blood count, × 10^9^/L	14.0(7.8)	14.1(6.3)	0.850	14.0(8.0)	14.1(6.3)	0.730
Hematocrit, %	43.2(10.8)	43.4(12.6)	0.786	43.1(9.4)	43.0(12.3)	0.978
Serum albumin, g/L	30.0(5.8)	31.8(7.0)	0.001	31.5(7.3)	31.7(7.3)	0.762
Serum urea, mmol/L	9.4(8.1)	7.7(7.2)	0.031	7.8(5.4)	7.6(6.2)	0.833
Serum creatinine, µmol/L	100.8(139.1)	83.4(78.6)	0.027	82.8(82.1)	80.5(60.1)	0.610
Serum calcium, mmol/L	1.7(0.4)	1.9(0.4)	0.003	1.8(0.4)	1.9(0.3)	0.974
Serum albumin _min_, g/L	27.0(4.0)	28.0(3.1)	0.002	27.4(3.0)	28.0(3.0)	0.168
SIRS score ≥ 2, n(%)	357(84.4)	109(74.7)	0.008	97(74.6)	98(75.4)	0.886
APACHE≥score ≥ 15, n(%)	109(25.8)	24(16.4)	0.022	27(20.8)	22(16.9)	0.428
IPN, n(%)	92(21.7)	7(4.8)	< 0.001	6(4.6)	7(5.4)	0.776
Persistent multiple organ failure, n(%)	149(35.2)	34(23.3)	0.008	27(20.8)	26(20.0)	0.878
Persistent multiple organ failure, n(%)						
Respiratory	380(89.8)	135(92.5)	0.350	124(95.4)	123(94.6)	0.776
Renal	137(32.4)	23(15.8)	< 0.001	20(15.4)	18(13.8)	0.726
Cardiovascular	78(18.4)	26(17.8)	0.865	17(13.1)	17(13.1)	1.000
Mechanical ventilation, n(%)	227(53.7)	37(25.3)	< 0.001	36(27.7)	34(26.2)	0.780
Hemofiltration, n(%)	76(18.0)	11(7.5)	0.003	11(8.5)	8(6.2)	0.475

IQR, Inter Quartile Range; BMI, Body Mass Index; Serum albumin _min_, the lowest albumin level within 1 week after admission; SIRS, Systemic Inflammatory Response Syndrome; APACHE, Acute Physiology and Chronic Health Evaluation; IPN, Infected Pancreatic Necrosis

**Table 5:** Comparison of death between with and without albumin infusions group

Outcome: death, n(%)	Hypoalbuminemia group		Normal group	
	PSM unadjusted	PSM adjusted	PSM unadjusted	PSM adjusted
With albumin infusions	131(31.0)	25(19.2)	48(19.4)	15(11.6)
Without albumin infusions	49(33.6)	41(31.5)	30(16.4)	24(18.6)
OR(95%CI)	0.89(0.60–1.33)	0.52(0.29–0.92)	1.22(0.74–2.02)	0.58(0.29–1.16)
*P*	0.562	0.023	0.430	0.121

### Subgroup analyses for hypoalbuminemia patients who received albumin infusions

To further examine the effect of albumin infusions for hypoalbuminemia patients with different doses within a week after admission and initial infusion time, two subgroup analyses according to whether the dose was greater than 100 g and whether the initial infusion time was greater than 48 h were applied. Similarly, PSM was used to minimize the influence of confounding factors in the two subgroup analyses, which were detailed in Supplementary Table S1 and Table S3. After PSM, a dose > 100 g within a week after admission for hypoalbuminemia patients with albumin infusions was associated with lower mortality than a dose ≤ 100 g (OR:0.51, 95%CI:0.28–0.90, P = 0.020) (Supplementary Table S2). However, there was no difference in whether the initial infusion time was ≤ 48 h (OR:0.86, 95%CI:0.46–1.60, P = 0.635) (Supplementary Table S4).

## Discussion

This retrospective research explores not only the relationship between albumin and the outcomes of SAP but also the association between albumin infusions and mortality in hypoalbuminemia patients with SAP. The lowest albumin level within 1 week after admission was associated with poor outcomes in SAP. After adjusting for some covariates, we found that a lower albumin level was independently associated with mortality. Furthermore, to further confirm the effect of albumin infusions in hypoalbuminemia patients who were grouped based on whether they had albumin infusions, PSM was conducted according to the serum albumin value and the disease severity to make sure that the two groups were comparable. This study demonstrated that albumin infusions could reduce the risk of mortality in hypoalbuminemia patients with SAP. Furthermore, infusing a sufficient albumin dose could decrease mortality for hypoalbuminemia patients with albumin infusions.

The early phase of AP usually takes place in the first week after disease onset and is characterized by SIRS and/or organ failure [5, 28]. The mechanism of hypoalbuminemia in AP may be driven by (1) a reduced intake of protein due to fasting and a high catabolism state in acute pancreatitis, (2) the fact that albumin is synthesized in the liver, and the excessive release of inflammatory cytokines such as IL-1, IL-6, and TNFα in SAP results in decreased liver synthesis, and (3) the increased vascular permeability increases the transcapillary loss of albumin in the progress of the stress response [26, 27, 34, 35]. Hypoalbuminemia may also occur when AP patients already exist malnutrition. These early complications were indeed associated with albumin consumption. This study demonstrated that the lowest albumin level within 1 week after admission was associated with poor outcomes for SAP patients. Therefore, the lowest albumin level within 1 week after admission could more accurately reflect AP progression and assess AP severity compared to the initial value of serum albumin. In addition, some studies have shown that the combined indicators related to albumin are more beneficial to predict the prognosis of AP patients than single indicators, such as the ratio of C-reactive protein (CRP) to albumin [36–39]. The level of CRP, as a positive acute phase reactant, will increases when infection or inflammation occurs [40]. Therefore, the CRP/albumin ratio will increase significantly when AP patients get worse. Similarly, it would be a good choice to analyze the highest CRP level of SAP patients within 1 week after admission, which can also more accurately reflect disease changes.

Early deaths might lead to lower hospital stays and costs. Because of the inclusion of patients who died within 30 days, and more of these patients were in the hypoalbuminemia group, which indicating actual differences between length of stay and costs to be even larger.

At present, there are many studies about the relationship between albumin infusions and the prognosis of critically ill patients. However, the effect of albumin infusions is still controversial. The SAFE study revealed that there was no significant difference in the 28-day mortality rate between the albumin group and the saline group. This subgroup analysis of severe sepsis patients showed only a decreasing trend of mortality for patients with albumin treatment (RR: 0.87, 95% CI: 0.74–1.02), which also had no significant difference [41]. A recent study of sepsis and hypoalbuminemia also showed that there was no significant difference in the 28-day mortality rate between the groups with and without albumin infusion [42]. In contrast, the Surviving Sepsis Campaign 2021 suggested using albumin in patients who received large volumes of crystalloids over crystalloids alone for adults with sepsis or septic shock [43]. In addition, the ALBIOS study reported that albumin infusion doesn’t improve the 28-day and 90-day mortality rates for sepsis patients. However, a post hoc subgroup analysis about septic shock patients showed a decrease in mortality among patients with albumin infusion (RR: 0.87, 95% CI: 0.77–0.99) [23], which is statistically significant. A systematic review reflected the same conclusion that albumin infusion significantly decreased the mortality of patients with septic shock [44]. The result is similar to the present study, which showed that there was a lower mortality among patients with an albumin infusion compared to patients without an albumin infusion (OR: 0.52, 95% CI: 0.29–0.92) for hypoalbuminemia patients with SAP. In general, albumin infusions are sufficiently safe, as few recent studies have shown that albumin infusions increase mortality. Multiple studies have shown that albumin infusions may decrease the mortality of patients with septic shock. This may be because SAP has many similarities to sepsis syndrome and septic shock [25]. Albumin infusions could also decrease the mortality for hypoalbuminemia patients with SAP due to the various physiological roles of albumin, such as maintaining an effective plasma colloid osmotic pressure, binding with endogenous and exogenous substances, scavenging free radicals as an antioxidant, causing anticoagulant effects, and maintaining the acid base status and anti-inflammatory effects [26, 45]. In addition, hypoalbuminemia has been associated with an intolerance to enteral feeding due to mucosal interstitial edema [46], so patients with albumin infusions had better tolerance to enteral feeding, leading to a higher daily caloric intake [27], which also contributes to disease recovery in SAP patients.

So far, there are few clinical trials that has explored therapeutic albumin infusions in SAP [19]. This is the first study to examined the association between albumin infusions and SAP, and this study can provide more guidance for the treatment of SAP. Since this was a retrospective study, serious bias existed in the severity among SAP patients regarding whether albumin was infused, and a strict PSM analysis was performed to decease the impact on confounding factors, such as disease complications and the albumin level, which might cause bias in the results. Through that analysis, we observed that albumin infusions may lead to obvious benefits, significantly reducing mortality in hypoalbuminemia patients with SAP.

This study further investigated the effect of albumin infusions for hypoalbuminemia patients with different doses by subgroup analysis, which showed that a dose > 100 g within a week after admission for hypoalbuminemia patients with albumin infusions was associated with lower mortality than a dose ≤ 100 g. The reason may be that adequate albumin infusion as a colloid is conducive to early fluid resuscitation to reduce mortality.

However, there are also some limitations in this study: (1) This is a retrospective cohort study, which may have selection bias. We increased the sample size as much as possible (N = 1000) and adopted strict inclusion and exclusion criteria to reduce bias. But using PSM reduced the sample size and the power of the study, which increased the probability of a type II error. (2) The period of data collection was too broad, spanning different economic conditions and medical standards, which would affect the use of albumin. (3) Other treatment methods, such as fluid administration, nutritional support and the use of blood plasma, also affect the serum albumin value and thus the albumin infusion. But these were not included in the study and not adjusted for. In the future, we should further design a quantitative study on energy supplement and hypoalbuminemia with SAP. (4) Since the data were collected in a large tertiary care hospital, they may not be generalizable to community or local hospitals because of different disease situation and medical resources.

## Conclusions

Our study revealed that the lowest albumin level within 1 week after admission was independently associated with mortality in SAP. In addition, infusing albumin may decrease mortality for hypoalbuminemia patients with SAP. In addition, infusing a sufficient albumin dose within a week after admission may decrease mortality for hypoalbuminemia patients treated with albumin infusions. Certainly, further prospective studies are required to confirm the clinical application of human serum albumin in SAP.

## Electronic supplementary material

Below is the link to the electronic supplementary material.

Supplementary Material 1

## Data Availability

All data generated or analyzed during this study are included in this published article (and its supplementary information files).

## Abbreviations

AP: Acute pancreatitis
SAP: Severe acute pancreatitis
BMI: Body mass index
serum albumin_min_: The lowest albumin level within 1 week after admission
SIRS: Systemic inflammatory response syndrome
APACHE: Acute Physiology and Chronic Health Evaluation
IPN: Infected pancreatic necrosis
ACS: Abdominal compartment syndrome
IL-1: Interleukin-1
IL-6: Interleukin-6
TNFα: Tumor necrosis factor α
CRP: C-reactive protein
