# Supplementary material for: Albumin infusion may decrease the mortality of hypoalbuminemia patients with severe acute pancreatitis: a retrospective cohort study
Source: BMC Gastroenterol. 2023 Jun 5;23:195. doi: 10.1186/s12876-023-02801-8 (PMC10243000; doi:10.1186/s12876-023-02801-8)
Supplement: Supplementary file 1 — Supplementary Material 1 [file 12876_2023_2801_MOESM1_ESM.docx]

**Table S1:** Baseline situation of the two groups with different doses within a week after admission after propensity matching analysis for hypoalbuminemia patients with albumin infusions

| **Variables** | **Before Matching** | | | **After Matching** | | |
| --- | --- | --- | --- | --- | --- | --- |
|  | **Dose＞100g** | **Dose≤100g** | ***P*** | **Dose＞100g** | **Dose≤100g** | ***P*** |
|  | **(N=149)** | **(N=274)** |  | **(N=101)** | **(N=101)** |  |
| Age, years, IQR | 50.0(21.0) | 53.0(19.0) | 0.030 | 51.0(25.0) | 51.2(18.0) | 0.826 |
| Sex (male), n(%) | 91(61.1) | 158(57.7) | 0.496 | 62(61.4) | 59(58.4) | 0.667 |
| BMI, kg/m^2^, IQR | 24.3(3.7) | 23.9(4.0) | 0.003 | 24.2(3.6) | 24.0(4.0) | 0.298 |
| Etiology, n(%) |  |  | 0.003 |  |  | 0.241 |
| Biliary | 58(38.9) | 152(55.5) |  | 47(46.5) | 43(42.6) |  |
| Hyperlipidemic | 70(47.0) | 87(31.8) |  | 41(40.6) | 37(36.6) |  |
| Alcoholic | 6(4.0) | 17(6.2) |  | 3(3.0) | 10(9.9) |  |
| Other | 15(10.1) | 18(6.6) |  | 10(9.9) | 11(10.9) |  |
| Hypertension, n(%) | 43(28.9) | 73(26.6) | 0.625 | 29(28.7) | 30(29.7) | 0.877 |
| Diabetes mellitus, n(%) | 27(18.1) | 32(11.7) | 0.068 | 14(13.9) | 14(13.9) | 1.000 |
| Laboratory data at admission, IQR |  |  |  |  |  |  |
| White blood count, × 10^9^/L | 14.0(7.6) | 14.0(8.0) | 0.974 | 15.0(7.9) | 14.0(8.4) | 0.807 |
| Hematocrit, % | 44.6(12.1) | 42.3(10.1) | 0.007 | 43.3(9.9) | 44.5(10.4) | 0.656 |
| Serum albumin, g/L | 29.1(4.6) | 30.8(6.5) | 0.001 | 28.8(5.2) | 30.0(6.8) | 0.257 |
| Serum urea, mmol/L | 11.7(9.4) | 8.6(6.8) | <0.001 | 10.0(9.7) | 10.0(8.2) | 0.441 |
| Serum creatinine, μmol/L | 153.4(200.5) | 84.3(93.2) | <0.001 | 113.9(159.9) | 119.1(157.1) | 0.421 |
| Serum calcium, mmol/L | 1.6(0.4) | 1.8(0.4) | <0.001 | 1.7(0.4) | 1.7(0.3) | 0.388 |
| Serum albumin _min_ , g/L | 28.0(3.2) | 27.0(3.1) | <0.001 | 27.4(3.1) | 27.0(3.0) | 0.065 |
| SIRS score≥2, n(%) | 133(89.3) | 224(81.8) | 0.042 | 89(88.1) | 90(89.1) | 0.825 |
| APACHEⅡscore≥15, n(%) | 51(34.2) | 58(21.2) | 0.003 | 28(27.7) | 29(28.7) | 0.876 |
| IPN, n(%) | 45(30.2) | 47(17.2) | 0.002 | 26(25.7) | 22(21.8) | 0.508 |
| Persistent multiple organ failure, n(%) | 77(51.7) | 72(26.3) | ＜0.001 | 40(39.6) | 48(47.5) | 0.256 |
| Persistent single organ failure, n(%) |  |  |  |  |  |  |
| Respiratory | 134(89.9) | 246(89.8) | 0.961 | 91(90.1) | 97(96.0) | 0.096 |
| Renal | 73(49.0) | 64(23.4) | <0.001 | 35(34.7) | 38(37.6) | 0.660 |
| Cardiovascular | 35(23.5) | 43(15.7) | 0.048 | 25(24.8) | 28(27.7) | 0.631 |
| Mechanical ventilation, n(%) | 111(74.5) | 116(42.3) | <0.001 | 65(64.4) | 68(67.3) | 0.656 |
| Hemofiltration, n(%) | 45(30.2) | 31(11.3) | <0.001 | 19(18.8) | 24(23.8) | 0.390 |

**Table S2:** Comparison of death between dose＞100g and dose≤100g within a week after admission for hypoalbuminemia patients with albumin infusions

| **Outcome: death, n(%)** | **PSM unadjusted** | **PSM adjusted** |
| --- | --- | --- |
| Dose＞100g | 52(34.9) | 30(29.7) |
| Dose≤100g | 79(28.8) | 46(45.5) |
| OR(95%CI) | 1.32(0.86-2.03) | 0.51(0.28-0.90) |
| P | 0.197 | 0.020 |

**Table S3:** Baseline situation of the two groups with different initial infusion time after propensity matching analysis for hypoalbuminemia patients with albumin infusions

| **Variables** | **Before Matching** | | | **After Matching** | | |
| --- | --- | --- | --- | --- | --- | --- |
|  | **≤48hours** | **＞48hours** | ***P*** | **≤48hours** | **＞48hours** | ***P*** |
|  | **(N=260)** | **(N=163)** |  | **(N=101)** | **(N=101)** |  |
| Age, years, IQR | 51.0(18.0) | 55.0(21.0) | 0.002 | 53.0(15.0) | 54.0(21.0) | 0.407 |
| Sex (male), n(%) | 153(58.8) | 96(58.9) | 0.992 | 60(59.4) | 58(57.4) | 0.775 |
| BMI, kg/m^2^, IQR | 24.2(3.9) | 23.9(4.0) | 0.145 | 24.0(3.7) | 23.9(4.3) | 0.878 |
| Etiology, n(%) |  |  | 0.001 |  |  | 0.582 |
| Biliary | 110(42.3) | 100(61.3) |  | 51(50.5) | 56(55.4) |  |
| Hyperlipidemia | 112(43.1) | 45(27.6) |  | 36(35.6) | 31(30.7) |  |
| Alcoholic | 18(6.9) | 5(3.1) |  | 7(6.9) | 4(4.0) |  |
| Other | 20(7.7) | 13(8.0) |  | 7(6.9) | 10(9.9) |  |
| Hypertension, n(%) | 68(26.2) | 48(29.4) | 0.460 | 18(17.8) | 25(24.8) | 0.229 |
| Diabetes mellitus, n(%) | 42(16.2) | 17(10.4) | 0.098 | 10(9.9) | 12(11.9) | 0.651 |
| Laboratory data at admission, IQR |  |  |  |  |  |  |
| White blood count, × 10^9^/L | 13.6(7.9) | 15.0(7.0) | 0.015 | 14.5(8.5) | 14.2(7.0) | 0.113 |
| Hematocrit, % | 43.6(12.0) | 42.8(8.7) | 0.318 | 43.5(11.9) | 42.8(9.2) | 0.320 |
| Serum albumin, g/L | 28.9(4.9) | 32.2(7.5) | <0.001 | 31.0(5.2) | 30.3(4.9) | 0.342 |
| Serum urea, mmol/L | 10.5(9.2) | 8.1(6.2) | <0.001 | 8.3(5.4) | 8.3(5.8) | 0.601 |
| Serum creatinine, μmol/L | 135.1(197.4) | 80.7(49.6) | <0.001 | 86.2(77.8) | 82.6(58.5) | 0.875 |
| Serum calcium, mmol/L | 1.7(0.4) | 1.9(0.4) | <0.001 | 1.8(0.4) | 1.8(0.4) | 0.762 |
| Serum albumin _min_ , g/L | 27.0(3.7) | 27.0(3.8) | 0.198 | 27.1(3.0) | 27.0(3.8) | 0.146 |
| SIRS score≥2, n(%) | 232(89.2) | 125(76.7) | 0.001 | 81(80.2) | 77(76.2) | 0.495 |
| APACHEⅡscore≥15, n(%) | 78(30.0) | 31(19.0) | 0.012 | 17(16.8) | 20(19.8) | 0.585 |
| IPN, n(%) | 60(23.1) | 32(19.6) | 0.403 | 20(19.8) | 25(24.8) | 0.398 |
| Persistent multiple organ failure, n(%) | 111(42.7) | 38(23.3) | <0.001 | 25(24.8) | 31(30.7) | 0.346 |
| Persistent single organ failure, n(%) |  |  |  |  |  |  |
| Respiratory | 237(91.2) | 143(87.7) | 0.257 | 91(90.1) | 94(93.1) | 0.447 |
| Renal | 106(40.8) | 31(19.0) | <0.001 | 17(16.8) | 21(20.8) | 0.471 |
| Cardiovascular | 58(22.3) | 20(12.3) | 0.010 | 15(14.9) | 19(18.8) | 0.452 |
| Mechanical ventilation, n(%) | 159(61.2) | 68(41.7) | <0.001 | 47(46.5) | 49(48.5) | 0.778 |
| Hemofiltration, n(%) | 65(25.0) | 11(6.7) | <0.001 | 9(8.9) | 10(9.9) | 0.810 |

**Table S4:** Comparison of death between initial infusion time ≤48 hours and＞48 hours for hypoalbuminemia patients with albumin infusions

| **Outcome: death, n(%)** | **PSM unadjusted** | **PSM adjusted** |
| --- | --- | --- |
| ≤48 hours | 96(36.9) | 26(25.7) |
| ＞48hours | 35(21.5) | 29(28.7) |
| OR(95%CI) | 2.14(1.36-3.36) | 0.86(0.46-1.60) |
| *P* | 0.001 | 0.635 |
